# PTM-SD: a database of structurally resolved and annotated posttranslational modifications in proteins

**DOI:** 10.1093/database/bau041

**Published:** 2014-05-24

**Authors:** Pierrick Craveur, Joseph Rebehmed, Alexandre G. de Brevern

**Affiliations:** ^1^INSERM, U 1134, DSIMB, F-75739 Paris, France, ^2^Univ Paris Diderot, Sorbonne Paris Cité, UMR-S 1134, F-75739 Paris, France, ^3^Institut National de la Transfusion Sanguine (INTS), F-75739 Paris, France and ^4^Laboratoire d'Excellence GR-Ex, F-75739 Paris, France

## Abstract

Posttranslational modifications (PTMs) define covalent and chemical modifications of protein residues. They play important roles in modulating various biological functions. Current PTM databases contain important sequence annotations but do not provide informative 3D structural resource about these modifications. Posttranslational modification structural database (PTM-SD) provides access to structurally solved modified residues, which are experimentally annotated as PTMs. It combines different PTM information and annotation gathered from other databases, e.g*.* Protein DataBank for the protein structures and dbPTM and PTMCuration for fine sequence annotation. PTM-SD gives an accurate detection of PTMs in structural data. PTM-SD can be browsed by PDB id, UniProt accession number, organism and classic PTM annotation. Advanced queries can also be performed, i.e*.* detailed PTM annotations, amino acid type, secondary structure, SCOP class classification, PDB chain length and number of PTMs by chain. Statistics and analyses can be computed on a selected dataset of PTMs. Each PTM entry is detailed in a dedicated page with information on the protein sequence, local conformation with secondary structure and Protein Blocks. PTM-SD gives valuable information on observed PTMs in protein 3D structure, which is of great interest for studying sequence–structure– function relationships at the light of PTMs, and could provide insights for comparative modeling and PTM predictions protocols.

**Database URL:** PTM-SD can be accessed at http://www.dsimb.inserm.fr/dsimb_tools/PTM-SD/.

## Introduction

The residues in a protein can undergo covalent and chemical modifications, which are usually called posttranslational modifications (PTMs). The concept of PTMs encompasses different types of modifications from a simple addition of atoms group such as the phosphorylation, e.g. done by Tyrosine kinase (1), to the binding of important large groups, e.g*.* the retinal in the bacteriorhodopsin (2). PTMs play important roles in modulating various biological functions by altering the physical and chemical properties, the localization and activity of proteins. They are also linked to multiple diseases, e.g*.* in nuclear receptors (3), in the regulation of metabolism (4) or in signal integration (5). Some modifications are specific to one kind of organism, as pupylation in prokaryotes (6), or particular types of residues, as mainly serine and threonine for O-linked glycosylation or S-nitrosylation for Cysteine.

The available data on PTMs increased drastically in the recent years because of the improvements of mass spectrometry-based detection methods (7). To simplify the analysis of complex PTM data and to enhance our understanding of various PTMs in different organism, many databases, software and tools have been developed. They are in general specific to some PTM types and/or specific to organism (8,9).

Recent studies have shown that PTMs have significant effects on the protein conformations and on their flexibility (10–12). Current databases contain crucial sequence annotation but do not provide valuable resource on the 3D structure related to these PTMs (13). In general, the available structural data refer to the protein chain for which PTMs are annotated, regardless of modifications are present in the solved structure. So far, few databases, such as dbPTM (14), PTMcode (15) and Phospho3D (16), use information from protein structures. dbPTM is an interesting database, which accumulates the biological information related to PTM, such as the catalytic sites, structural information, solvent accessibility of residues, protein secondary structures, protein domain and protein variations (14). The main objective of dbPTM is to summarize all experimental information on PTM as sequence analysis and prediction methodology. PTMcode presents the functional associations between 13 different PTM types within proteins in 8 eukaryotic species (15). The structural data consists of mapped PTM residues to 3D structures of proteins from the Protein Data Bank (17), which do not always correspond to modified residues. Phospho3D, as many databases, is specific to one type of PTM and provides 3D structures of phosphorylation sites according to the annotations of the phospho.ELM database (18). The database also collects the results of a large-scale structural comparison procedure providing clues for the identification of new putative phosphorylation sites.

Current databases do not underline PTMs in the protein structures context owing to the difficult identification of resolved PTMs in the PDB files. Posttranslational modification structural database (PTM-SD) provides access to structurally solved modified residues, which are also experimentally annotated as PTMs. It gives valuable information on PTMs in the context of global and local protein conformation (19), and also particular details for each PTM observed in protein 3D structure. The proper availability of these data could be of great interest for studying sequence–structure–function relationships at the light of PTMs, which are often forgotten. It could also be useful and provide insights for comparative modeling and PTM predictions protocols.

PTM-SD can be accessed at http://www.dsimb.inserm.fr/dsimb_tools/PTM-SD/.

## Materials and Methods

**Figure 1.**
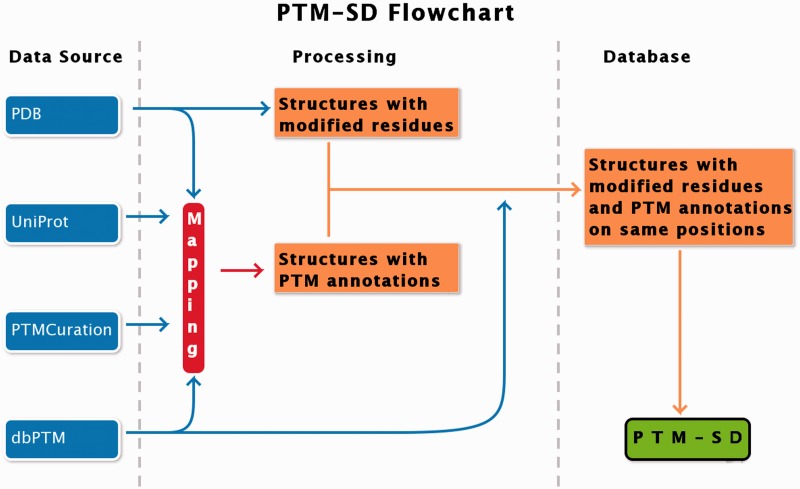
PTM-SD flowchart. Four different databanks are used to generate the data. The protein structures are taken from PDB (17), while PTMs annotations are extracted from dbPTM (14) and PTMCuration (20). UniProt sequences (21) are aligned against the extracted PDB sequences. Thus, we obtained protein structures with PTM annotations and modified residues at exact same positions. At last a semantic mining was made to accept or not the correspondence between the modifications and the annotations.

PTM-SD entry consists of structurally resolved and experimentally annotated PTMs in proteins structures. Flowchart in Figure 1 explains the principles of the database. The major problem encountered in building this database was the detection of PTMs in the PDB structures. At this time, no efficient query can be done on RCSB Protein Data Bank (17) to select easily and exclusively protein structures containing solved PTMs. Searching for specific PTM keywords could lead to structure of enzymes implicated in modifications. By using the advanced search, the RCSB PDB returns, as on 22 January 2014, a total of 17 767 structures of proteins, peptides and protein/nucleic acid complexes containing at least one modified residue. These modified residues are not all related to PTMs, and could correspond to protein engineering.

To build our database, we proceed step-by-step. Firstly, we selected protein structures containing modified residues (indicated in PDB files with the MODRES records) with available PTM annotations. These last were extracted from dbPTM (14) and PTMCuration (20). Only experimentally verified annotations were selected.

Secondly, we verified, in the selected structures, that the numbering of modified residues corresponds to the position of annotations in sequence. Residue numbering from PDB does not necessarily follow the amino-acid positions in the corresponding protein sequence. The first idea was to extract the sequence from PDB and align it with the UniProt sequence. The extraction of sequence from PDB could be difficult for many reasons: numbering in PDB may not be continuous because of the insertion of residue (numbering for example 100-100A-100B-103) or insertion of protein fragment (like lysozyme in PDB structure 2RH1), the structure could contain missing residues, gaps (but not necessarily along with gaps in numbering), mutations could be present and the residue names could be different from the 20 standard amino acids. Additionally, different PTMs can be annotated to the same sequence position; therefore, some disagreements between the structure of the PTMs in the PDB and annotations in dbPTM were observed (see the example ‘N-trimethyllysine’ in position 4 of PDB 3N9L chain B, which is also found annotated as ‘N6-acetyllysine’. http://www.dsimb.inserm.fr/dsimb_ too ls/ PTM-SD/ request_ details.php?pdb_id=3N9L&p db_ ch ain=B&pdb_pos=4).

Finally, the last step was to deal with ‘incompatibilities’ encountered between PTMs annotations, MODRES PDB records and chemical structures of the modified residues. PTMs are associated with a large variety of chemical groups and can be described with unusual residue names and atom names. Based on the wwPDB Format Documentation (the Contents Guide Version 3.30, 21 November 2012) and the Section A, wwPDB processing procedures (January 2014 Version 2.7), a modified residue is annotated in MODRES record and could be described in two ways. If the modification was made by a chemical group greater than 10 atoms, the modified residue will be split into two groups: the amino acid atoms (defined in ATOM record) and the modification atoms (defined in HETATM record) grouped in a ‘hetero group’ and indicated in HET records. The covalent bond between these two groups is indicated in the LINK record. In a second way, if the modification involves 10 atoms or lower, all the atoms are grouped into a ‘hetero-group’.

For example, in the Chain B of PDB 1J2E, the Asn 85 is glycosylated (N-linked). As seen at the bottom of its ‘detail page’ (http://www.dsimb.inserm.fr/dsimb_tools/PTM-SD/request_ details.php?pdb_ id=1J2E&pdb_chain= B& pdb_ pos =85), the coordinate information of the atoms of Asn 85 are given in the ATOM PDB record, and all the coordinate information of the sugar are indicated in HETATM PDB record.

In a different way, the Thr 160 of the chain C in the PDB 2UZB is annotated as phosphorylated (see http://www.dsimb . inserm.fr/dsimb_tools/PTM-SD/ request_ det a ils. php?pdb_ id=2UZB&pdb_chain= C&pdb_pos=160). In MODRES record, the residue name THR are replaced by TPO and all the coordinate information are given in the HETATM record, grouping together the phosphate atoms (P/OP1/OP2/OP3), the side chain atoms (CB/OG1/CG2) and the backbone atoms (N/CA/C/O). In this case, the covalent links indicated in LINK records refer to the polypeptide links with the previous and next residues in the polypeptide chain.

Hence, the consistency of these three information (PTMs annotations, MODRES PDB records and chemical structures) was checked by automatic and manual refinement, using extracted data from the PDB files and a correspondence annotation table (http://www.dsimb.inserm.fr/dsimb_tools/PTM-SD/correspondence_table.html).

In summary, a mapping was made, through the UniProt accession number (Uniprot AC) (21), between the annotation databases (PTMCuration, dbPTM) and the PDB. The obtained structures were automatically and manually inspected to validate the annotations in terms of position and chemical structure.

Extraction of structural data from PDB file (as sequence, missing information) was done with in-house scripts. Secondary structures were assigned using the most popular method, DSSP (22). It assigns the secondary structures by particular hydrogen-bond patterns detected from the protein geometry and an electrostatic model. DSSP is the tool used by the PDB to assign secondary structure. It defines three kinds of helices (α, π and 3_10_), β-sheet, β-bridge, two kinds of turns (hydrogen-bonded turns and non–hydrogen-bonded bend) and the remaining is considered as a coil. Next, we assigned Protein Blocks (PBs) (23). This structural alphabet is composed of 16 local structure prototypes of five residues in length. They efficiently approximate every part of protein structures. The PBs *m* and *d* can be roughly described as prototypes for the central region of α-helix and β-strand, respectively. PBs *a* through *c* primarily represent the N-cap of β-strand, whereas *e* and *f* correspond to C-caps; PBs *g* through *j* are specific to coils, PBs *k* and *l* correspond to N cap of α –helix and PBs *n* through *p* to C-caps. They have been used in various approaches, for example in protein superimposition (24,25), and for the analysis (26,27) or prediction (28,29) of protein binding sites. PB assignment was done with a slightly modified Python PBxplore tool (https://github.com/pierrepo/PBxplore).

The equivalent number of PBs (*N_eq_*) is a statistical measurement similar to an entropy and represents the average number of PBs a given residue takes (23). *N_eq_* is calculated as follows:

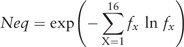

Where *f*_x_ is the observed frequency of PB *x*. A *N_eq_* value of 1 indicates that only one type of PB is observed, while a value of 16 is equivalent to a random distribution.

The 3D structure representation is generated using PyMOL software (http://www.pymol.org).

## Results

### Statistics

On 22 January 2014, PTM-SD consisted of 10 628 entries. It corresponds to 842 Uniprot AC, 2986 PDB files and 5350 PDB chains containing at least one modified position. Twenty-one different kinds of PTMs were detected, 11 with >50 occurrences (see Table 1). As expected, the most important one is glycosylation (60.09%), followed by phosphorylation (15.23%) and methylation (8.10%). Two hundred six different organisms are present, with an overrepresentation of Human (44.27%), and other mammals, e.g. mouse (7.98%), bovine (7.79%), pig (1.64%) and rat (1.52%). Green alga (5.84%) and chicken (2.71%) are also well represented.

**Table 1.** Distribution of the 21 kinds of PTMs in PTM-SD

PTM	Frequency	Percentage (%)
N-linked glycosylation	6386	60.09
Phosphorylation	1619	15.23
Methylation	861	8.10
N6-carboxylysine	390	3.67
Hydroxylation	314	2.95
Pyrrolidone carboxylic acid	308	2.90
O-linked glycosylation	204	1.92
Gamma-carboxyglutamic acid	195	1.83
Formylation	131	1.23
Acetylation	93	0.88
Oxidation	57	0.54
Sulfation	24	0.23
S-Nitrosylation	17	0.16
Pyridoxal phosphate	15	0.14
TPQ	4	0.04
LTQ	2	0.02
Pyruvate	2	0.02
Lipoyl	2	0.02
Retinal protein	2	0.02
Nitration	1	0.01
Bromination	1	0.01

In terms of secondary structure assignment, PTMs are mainly observed in loops or irregular secondary structure (36.41%). Surprisingly, the turns (hydrogen and non-hydrogen bonded) are highly overrepresented (30.68%), whereas the classical α helix is seen only 11.18%, which is low (the average frequency of α helix in proteins is 30%).

PTMs in structural data are not precisely recorded, making their identification difficult (see ‘Material and Methods’ section). Discordance between UniProt position and PDB residue numbering is observed in 51% of PTM-SD entries. The atom coordinates, backbone atoms included, of 27% of PTM-SD entries are recorded in the HETATM PDB records, which are usually reserved for molecules that are not part of a biological polymer (as prosthetic groups, inhibitor, solvent molecules and ions). As far as, in PTM-SD, 38% of entries described in PDB files have a different three-letter code known for the 20 classical amino acids; e.g. threonine known as THR is named TPO in case of phosphorylation, or lysine known as LYS is defined as M3L in case of trimethylation.

### Browse PTM-SD

#### General search

**Figure 2.**
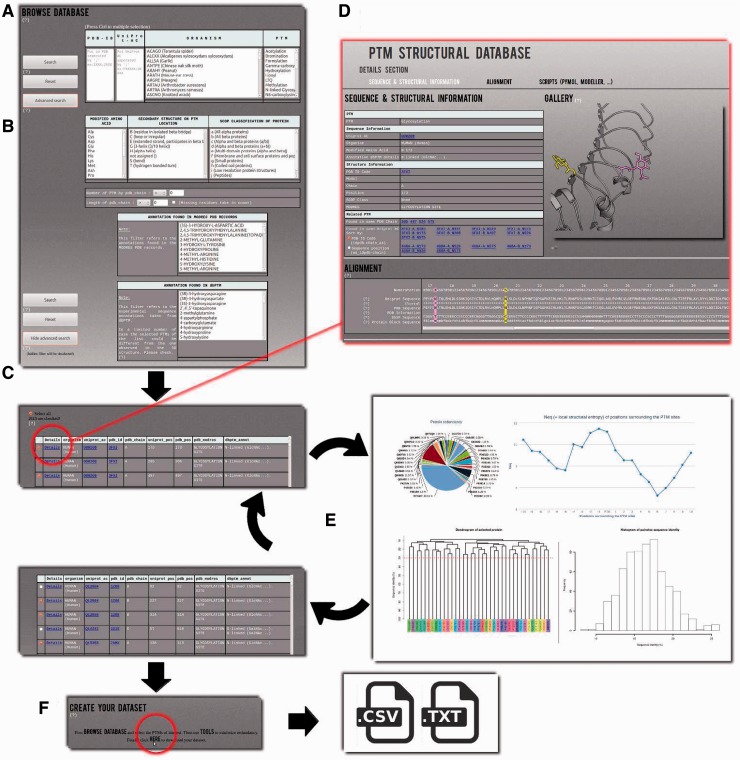
Example of PTM-SD usage. (**A**) A simple mode is available to use PTM-SD. It is possible to look for a list of PDB and/or UniProt ids, combined with a specific organism and (a) particular PTM(s). (**B**) An advanced mode allows more complex requests, as specific amino acid(s), secondary structure(s), SCOP fold(s), number of PTM by PDB chain, length of PDB chain and detailed annotation(s) found in PDB records and dbPTM. By clicking on the search button, (**C**) a results table appears. It gives for each entry the information on organism, cross-linking with the PDB and UniProt id, the precise position of the PTM in sequence and structural data, PTM annotations and its structural environment in terms of PBs and secondary structures. (**D**) By clicking on the ‘details’ link, the visitor is redirected to a new page containing extra information on the selected PTM site. Related PTM-SD entries found in same PDB chain and same UniProt AC are accessible through cross-link. Image gallery was done, thanks to PyMOL software, and below alignment section, done thanks to Clustal W, allows direct observation of the sequence/structure relationship surrounding PTM sites. On this page, scripts for PyMOL software and template sequence for Modeller comparative software are provided. (**E**) From the complete set of selected entries, it is possible to look at the distribution of organisms, proteins, PDB ids/chains and PTM types. Neq entropy index quantifies the local structural divergences between the PTMs. From this step, the visitor can reduce the protein redundancy into his selected entries. (**F**) At last he can download the list of PDB chain and the PTM-SD data related to his selection.

The data can be explored using two search modes: (i) the simple mode (Figure 2A) and (ii) the advanced mode (Figure 2B). The first one allows searching the PTMs by PDB id(s), and by UniProt accession number(s) and then filtering according to the classic PTM annotation (21 different kinds of PTMs are proposed at this level), and specific organisms; both short name and more common one are given, e.g. ACAGO (*Tarantula spider*). Multiple choices can be done, and all criteria can be combined to complete more complex queries. The advanced mode adds more precise criteria. A dedicated research can be done with selecting specific amino acid types, secondary structures, SCOP class classification (30), protein length, as well as number of PTMs observed in PDB chain. Additionally two detailed annotations are provided, which came directly from the PDB MODRES records and dbPTM fields. The results are provided as a Table under the search area (Figure 2C) in which each line corresponds to one PTM-SD entry, i.e. one PTM experimentally annotated and structurally solved.

#### Details

The results are given in the table, for each line, the organism, the UniProt AC, the PDB and chain identifiers. Additionally, the specific annotation found in MODRES PDB record and the dbPTM annotation(s) with their corresponding position are provided, as they can differ. For instance, the methylation observed on residue 4 in the histone demethylase ceKDM7A [PDB code 3N9L chain B, (31)] is defined in the MODRES PDB record as ‘N-TRIMETHYLLYSINE’. At the corresponding position in the protein sequence (Lysine 5 in P08898), four different annotations are found in dbPTM: N6-methyllysine, N6, N6-dimethyllysine, N6,N6,N6-trimethyllysine and N6-acetyllysine.

The SCOP class of the PDB chain, the amino acid corresponding to the PTM site position in the protein sequence and the assignments of PBs and secondary structure in the vicinity of the PTM site are also given. The PTM site is colored in purple to locate it easily. For each entry, cross-links to PDB and UniProt Web sites are provided. Finally, a ‘details’ link for each entry is provided. By clicking on it, users will be redirected to a dedicated webpage (Figure 2D) where all PTMs annotations found in dbPTM related for this specific position are available. Related PTM-SD entries are given for other positions on the protein chains and for the same UniProt AC. Sometimes, a huge number can be found, *like* the glycosylation observed on position 150 in the chain A of the human dipeptidyl peptidase IV [PDB code 1TK3 (32)], which has six related PTM-SD entries on the same chain and 667 on the same UniProt AC.

In the same, below the various information, the visitor can find useful aligned information, which allows the analyses of PTMs in the sequence and structural context simultaneously. An alignment between the sequence from protein structure and the sequence from UniProt was performed by ClustalW 2.0.12 (33) and symbols for similarity/identity of positions are showed. A sequence representing the extracted information from PDB records is also aligned emphasizing discrepancies, missing residues, inserted residues and residues for which all the atoms are defined in HETATM records. Additionally, sequences of secondary structure and PBs assignments are also aligned.

On the current ‘details’ page, the selected PTM position is highlighted in purple, whereas the other PTM sites found in the PDB chain are colored in yellow. The PTM position found in other PDB chain are highlighted in green. To help the visitor to explore the 3D structure, the PyMOL script used to compute the PTM gallery is also provided. Please note that the PTMs are highlighted in the same color as in the aligned information. Finally, extracted data are given from the PDB file, corresponding to the modified residues, and a sequence that could be directly used to perform comparative modeling with Modeller software (34).

#### PTM-SD tools/specific dataset creation

After selecting entries from the results table, the visitor has access to three tools (namely ‘Statistics’, ‘Neq’ and ‘Clustering’) developed to make a quick analysis of the PTMs selection and to facilitate the creation of specific PTMs dataset (Figure 2E). For instance, with the simple and advanced search, it is easy to define a set of protein structures. By using the ‘Statistics’ tool, the visitor can analyze the distribution of a PTM with regards to an organism, e.g. ‘Methylation’ is found in structures from 24 organisms, with *Chlamydomonas reinhardtii* being the most important one (33.91%). Similarly, the visitor can look at an organism and see which PTMs are found, e.g. three different PTMs are observed in rabbit proteins (Methylation, Phosphorylation and N-linked Glycosylation). By selecting ‘Methylation’ and ‘Rabbit’, 67 entries are found. The ‘Statistics’ tool shows that in fact these entries correspond to one annotation of methylation found in one UniProt AC, but solved in 35 PDB id codes and 67 PDB id chains. This methylation does not belong to only one local conformation, as showed by the ‘Neq’ tool, which is an entropy index based on the PB assignment at the vicinity of the PTMs site. At one position, if all the PBs are identical, *Neq* equals to 1, whereas if the 16 are seen equivalently, *Neq* equals to 16. For this PTM site, *Neq* equals to 1.79, underlining differences between local conformations; surprisingly, it is before the PTM that *Neq* equals to 1. At last, by using ‘Clustering’ tool, the visitor has the possibility to compute dendogram and histogram of pair-wise sequence identity between selected proteins. He could also reduce redundancy in his selected entries, thereby creating a nonredundant selection. For instance, the ‘Hydroxylation’ represents 34 UniProt AC for 64 PDB id codes, 165 PDB id chains and 314 PTM-SD entries. The use of a 30% threshold leads to deselect entries from the results table (Figure 2E). The related PTM-SD data and the list of PDB ids can be downloaded at the ‘create your dataset’ section (Figure 2F).

These data could be of great interests for studying sequence–structure–function relationships at the light of PTMs. For example, users interested in studying the impact of phosphorylation in protein structure could easily browse the database and get access to all the phosphorylated residues solved in structure and experimentally annotated. By using the ‘Neq tool’ they could have a quick first analysis of the local conformation observed at the neighboring phosphorylation site. This type of analysis could be easily done again withmore precise criteria, as specific organisms or specific modified amino acids.

Then by using the ‘Clustering tool’ they could create, and later download, a nonredundant data set, which will be the base of work for extraction of structural descriptors needed in a classic phosphorylation prediction protocols.

Similarly, users motivated by comparative modeling could use the database to select protein structures associated with specific experimentally annotated PTMs. By using the extracted data from PDB, and the clean sequence given for Modeller software, which are available in ‘detail page’ of each entry, they have the necessary data to compute protein model containing PTMs.

#### Implementation

PTM-SD is developed and maintained using MySQL. All data extraction, treatment and update are carried out with Python scripts. The front-end interface was developed in HTML/PHP and animated using JavaScript/jQuery. The interactive graphics and flowcharts are generated using the JavaScript charting library Highcharts 3.0 (www.high charts.com). The clustering is computed using ‘hclust’ and ‘cutree’ functions of the R statistical software, version 2.15 (http://cran.r-project.org/). All gallery illustrations were rendered using the molecular visualization software PyMOL 1.5.

#### Update

PTM-SD is regularly updated in two steps. The structural data are updated weekly, immediately after the PDB updates, and the annotations data are updated each month.

## Discussion

Structural data are limited in PTMs databases, e.g. in dbPTM (14), Phospho3D (16) or PTMcode (15). It mainly corresponds to secondary structure or solvent accessibility predictions. The available 3D representation of proteins is present only for visualization purposes. It facilitates the investigation of structural characteristics surrounding the PTM sites regardless to the real presence of the annotated PTMs. Other databases give access to the structure of the modification itself, but out of the protein fold context, e.g. RESID (35), BCSDB/Glycoscience (36) or GlycomeDB (37). Some databases provide cross-linking or id mapping from the PDB where annotated PTMs are resolved; however, they are specific to one PTM type of organism, e.g. Glycan Fragment DB (38), O-GLYCBASE (39) and ProGlycProt (40).

PTM-SD is the only structural database that is generic to numerous PTMs in PDB structures and also underlines the complex case of many PTMs, i.e. structure discrepancies or multiple PTMs annotation. It was designed as an easy-to-use tool to meet the needs of different scientific communities: for biologists interested by PTM data for specific proteins, or organisms, and for bioinformatician searching for structural criteria to take into account in structural studies or in modeling/prediction protocols.

Our extensive analyses of structural data had revealed that (i) the number of PTMs in protein structures is not negligible, (ii) some PTMs are heterogeneous and difficult to access properly in the PDB files because they are not really protein materials, (iii) the annotation of the same modification can be different and (iv) more surprisingly, a PTM can be crystallized in the structure while annotated as a different type of PTM in other databases. PTM-SD gives an overview of the ensemble.

In the future, it is planned to keep developing PTM-SD by adding filters related to structural data, as resolution, R-Factor, B-Factor, solvent accessibility, by giving direct access to the coordinate data of PTM atoms, and by providing other options for the clustering tool.
